# Association of several loci of SMAD7 with colorectal cancer: A meta-analysis based on case–control studies

**DOI:** 10.1097/MD.0000000000032631

**Published:** 2023-01-06

**Authors:** Qiang Xiao, Jian Chen, Jia Zhu, Shukun Zeng, Hu Cai, Guomin Zhu

**Affiliations:** a General Surgery Department, First Affiliated Hospital of Nanchang University, Jiangxi, China.

**Keywords:** bioinformatics analysis, colorectal cancer, meta-analysis, SMAD7, SNPs

## Abstract

Sma-and mad-related protein 7 (SMAD7) can affect tumor progression by closing transforming growth factor-beta intracellular signaling channels. Despite the extensive research on the correlation between SMAD7 polymorphisms and colorectal cancer (CRC), the conclusions of studies are still contradictory. We conducted a study focusing on the association of SMAD7 polymorphisms rs4939827, rs4464148, and rs12953717 with CRC.

**Methods:**

We searched through 5 databases for articles and used odd ratios (ORs) and 95% confidence intervals (CIs) to discuss the correlation of SMAD7 polymorphisms with CRC risk. The heterogeneity will be appraised by subgroup analysis and meta-regression. Contour-enhanced funnel plot, Begg test and Egger test were utilized to estimate publication bias, and the sensitivity analysis illustrates the reliability of the outcomes. We performed False-positive report probability and trial sequential analysis methods to verify results. We also used public databases for bioinformatics analysis.

**Results:**

We conclusively included 34 studies totaling 173251 subjects in this study. The minor allele (C) of rs4939827 is a protective factor of CRC (dominant, OR/[95% CI] = 0.89/[0.83–0.97]; recessive, OR/[95% CI] = 0.89/[0.83–0.96]; homozygous, OR/[95% CI] = 0.84/[0.76–0.93]; heterozygous, OR/[95% CI] = 0.91/[0.85–0.97]; additive, OR/[95% CI] = 0.91/[0.87–0.96]). the *T* allele of rs12953717 (recessive, OR/[95% CI] = 1.22/[1.15–1.28]; homozygous, OR/[95% CI] = 1.25/[1.13–1.38]; additive, OR/[95% CI] = 1.11/[1.05–1.17]) and the *C* allele of rs4464148 (heterozygous, OR/[95% CI] = 1.13/[1.04–1.24]) can enhance the risk of CRC.

**Conclusion:**

Rs4939827 (T > C) can decrease the susceptibility to CRC. However, the rs4464148 (T > C) and rs12953717 (C > T) variants were connected with an enhanced risk of CRC.

## 1. Introduction

Colorectal cancer (CRC) is a global disease with a high incidence and death rate, ranking fourth and fifth among all malignancies.^[1]^ Hereditary genomic alterations have been linked to a person’s chance of acquiring cancer for decades.^[2]^ As a result, research into the genetic variables that influence CRC susceptibility is critical. Sma-and mad-related protein 7 (SMAD*7*), as a member of the SMAD family, may play a damaging role in the transforming growth factor-beta (TGF-*β*) signaling pathway by interacting with the mobilization of other SMAD proteins, which indirectly contributes to tumorigenesis.^[3]^ As a critical component in the intracellular signaling shutdown mechanism of transforming growth factor (TGF), it can block the tumor-suppressive function of TGF-*β* at an early stage of carcinogenesis, such as CRC.^[4]^

The TGF-*β* family is strongly associated with development and endocytosis in most tissues. TGF-*β* family members bind to type II and type I receptors, forming a receptor complex that phosphorylates type I receptors, initiating TGF-*β* signaling and phosphorylating the made-associated proteins SMAD2 and SMAD3. Later on, phosphorylated SMAD2 and SMAD3 bound to SMAD4 to forge the SMAD4- RSmad complex, which then enters into the nucleus and regulates the transcription of specific target genes with the help of DNA-binding protein chaperones.^[4–6]^ TGF activation inhibits TGF-mediated phosphorylation of SMAD2 and SMAD3 since SMAD7 may connect to the TGF receptor complex but is not phosphorylated.^[5]^

All 3 polymorphic variants occur on chromosome 18. rs4939827, rs464148 and rs12953717 are located within intron 3 of SMAD7 on chromosome 18q21.^[7]^ Rs4939827 and rs12953717, as 2 adjacent polymorphisms, do not have the same direction of base mutation. Rs4939827 mutates from base *T* to base *C*. In contrast, rs12953717 mutates from base *C* to base *T* to affect gene expression. rs4464148 has the same direction of base mutation as rs4939827. This irreversible mutation alters the protein structure at the molecular level and thus affects biological function.^[8]^

Since its discovery, SMAD7 has been widely researched, especially to study its single nucleotide polymorphisms with cancer because of its possible signaling inhibition in the cell nucleus. We found that rs4939827, rs4464148, and rs12953717 polymorphisms were studied more in association with various cancer, which contains colorectal, breast, hepatocellular carcinoma, esophageal, chronic lymphocytic leukemia.^[9–12]^ Among them, CRC is the most. However, many studies have conflicting results. Some studies concluded that SMAD*7* variants are not significantly associated with CRC.^[13–15]^ but most of the results indicated the increased risk.^[16–18]^ The inconsistent results might be attributed to a small sample or chance error. Therefore, we included studies of these 3 variants with CRC, providing a more adequate and accurate study of the correlation between SMAD7 polymorphisms and CRC.

## 2. Materials and methods

Our research has been registered in PROSPERO and the details are available at https://www.crd.york.ac.uk/PROSPERO/. Since we are not engaged in human or animal experiments, we do not have to submit an ethical application. Our study was closely carried out according to the PRISMA guidelines.

### 2.1. Search strategy

Studies included in this meta-analysis that met the inclusion criteria were obtained from 5 databases (PubMed, Embase, Web of Science, Wan Fang database, and China National Knowledge Infrastructure), with a search time limit of literature published earlier than January 2022. The search strategy incorporated these terms: “Neoplasm,” “tumor,” “cancer,” “malignancy,” “colorectal cancer,” “CRC,” “SNP,” “Polymorphism,” “mutation,” “SMAD7,” “SMAD7 protein,” “rs4939827,” “rs4464148,” “rs12953717,” “case–control study”; the specific search formula can be viewed in the supporting information (See Table S1, Supplemental Digital Content, http://links.lww.com/MD/I297, which shows detailed search strategies). We also reviewed articles about our study by the references cited in the included studies; if feasible, gray literature searched via manual was also included. Two researchers independently searched through the above search strategy and deliberated on inconsistent search results. If necessary, we need a third researcher join us until reaching a consensus. There were no language constraints in this search.

### 2.2. Inclusion and exclusion criteria

Two researchers searched and filtered separately in the specified databases under the same strategy. If discrepant results arose, discussions were held, and a third researcher joined when needed until a consensus was reached.

#### 2.2.1. Inclusion criteria.

Studies can be included only by meeting the following inclusion criteria:
(a) Case–control studies.
(b) evaluating the correlation between SMAD7 loci (rs4464148, rs12953717, rs4939827) and CRC risk, participants in the case group must have malignancy confirmed by pathological methods.
(c) Full text, acquired genotype frequencies existed in both the case and control groups.

#### 2.2.2. Exclusion criteria.

1. Duplicate literature.
2. Non-human experiments. In multiple studies with overlapping, duplicate data published, only the most recent or intact study was included.

### 2.3. Data extraction

The 2 researchers respectively derived some contents from available pieces of literature: first author, country region, year of publication, ethnicity, source of the control group, cancer type, and genotype frequency of case and control groups, genotyping method. The 2 researchers cross-checked the extraction data to avoid any discrepancies. Genotype frequencies in the control group must follow Hardy-Weinberg equilibrium (HWE). HWE for each study was measured by *χ*2 test, and *P* > .05 was consistent with HWE. We will exclude trials that do not conform to HWE.

### 2.4. Quality assessment

Two investigators assessed the value of included studies under the Newcastle-Ottawa scale, a total score of 9 points. Case–control trials were scored in 3 dimensions: selection, comparability, and exposure. Scores of 5 to 9 were categorized as high quality versus scores of 0 to 4, which were considered low quality (See Table S2, Supplemental Digital Content, http://links.lww.com/MD/I298, which show the results of literature quality evaluation). In case of differences between the 2 investigators, it was necessary to discuss with a third party until the 3 parties reached an acceptable resolution.

### 2.5. Statistical analysis

software: Stata 15.1 (http://www.stata.com), trial sequential analysis (TSA) 0.9.5.10 Beta.

#### 2.5.1. Meta-analysis.

The pooled odd ratios (ORs) and 95% confidence interval (CIs) were taken to evaluate the relationship between the 3 polymorphisms and CRC in dominant, recessive, homozygous, heterozygous and additive models. We compared the relationship between the value of OR and 1.0 and whether the value 1.0 is in the 95% CI. If the 95% CI contains the value 1.0, then the results will be deemed insignificant.

#### 2.5.2. Heterogeneity analysis and subgroup analysis.

We used Cochran *Q* test and I-square to assess the level of primary studies’ heterogeneity. a *P*-value of less than 0.1 or an I-square greater than 50% for the Cochran *Q* test was defined as significant heterogeneity. Fixed-effects models were adopted to pooled ORs to estimate the relationship between respective models and CRC risk in case of insignificant heterogeneity; otherwise, random-effects models were adopted. We performed a Meta-regression analysis to probe the root of heterogeneity. We conducted subgroup analyses of included studies for ethnicity, sample, and source of control group to further probe the sources of heterogeneity.

#### 2.5.3. Publication bias.

We employed contour-enhanced funnel plots, Begg test, and Egger test to evaluate the risk of publication bias. The publication bias existed if the funnel plot was asymmetric in the white region or the *P*-value of the Begg test and Egger test was less than .05.

#### 2.5.4. Sensitivity analysis.

Since we included more than 10 studies in each polymorphism, the reliability of the results for single nucleotide polymorphisms could be assessed by the leave-1-out method. We sequentially excluded a case–control study, and the leftover studies were subjected to sensitivity analysis to examine whether there was a discrepancy between the results of the excluded options and the primitive overall result. Some studies may be considered for exclusion if the study statistics compromise the reliability of the results.

### 2.6. Reliability assessment

We employed the false-positive report probability (FPRP) method for statistical indicators of positivity, which detects the occurrence probability of type 1 errors caused by cumulative meta-analysis. We set the threshold for FPRP at 0.2, calculated statistical power at an odd ratio of 1.5, and allocated a prior probability of 0.1. Results will be considered significant if the FPRP calculated by statistical power, prior probability, and the *P*-value is less than .2.^[19]^ In addition, we also performed the test sequential analysis strictly according to the TSA user manual^[20]^ and using the latest software (TSA 0.9.5.10 Beta, www.ctu.dk/tsa). All of them are available at www.ctu.dk/tsa. We set the probability of a type 1 error to 0.05, a power of 80%, and a conventional bound of 1.96 (Z = 1.96). The allele models for the 3 polymorphisms were analyzed, and we will consider the inclusion of sample size sufficient when the cumulative *Z* value crosses the monitor boundary or when the sample size is greater than the required information size.

### 2.7. Bioinformatics analysis

#### 2.7.1. Protein interactions network analysis.

We use an open-source data site STRING (https://cn.string-db.org/) to analyze SMAD7 concerning its human protein interactions,^[21]^ with data derived from high-throughput experimental data, computer genome prediction, and automated text mining data. In addition, it can also visualize the data into an intelligible gene co-expression network.

#### 2.7.2. Enrichment analysis.

The DAVID database (https://david.ncifcrf.gov/) is a bioinformatics database that integrates biological data and analytical tools to provide systematic and comprehensive biofunctional annotation information for the large-scale gene or protein lists.^[22]^ We obtained functional annotation information of SMAD7 co-expressed proteins from it and visualized the data in the bioinformatics platform (https://www.bioinformatics.com.cn/). A false discovery rate less than 0.05 was statistically significant.

## 3. Result

### 3.1. Screening process

**Figure 1.**
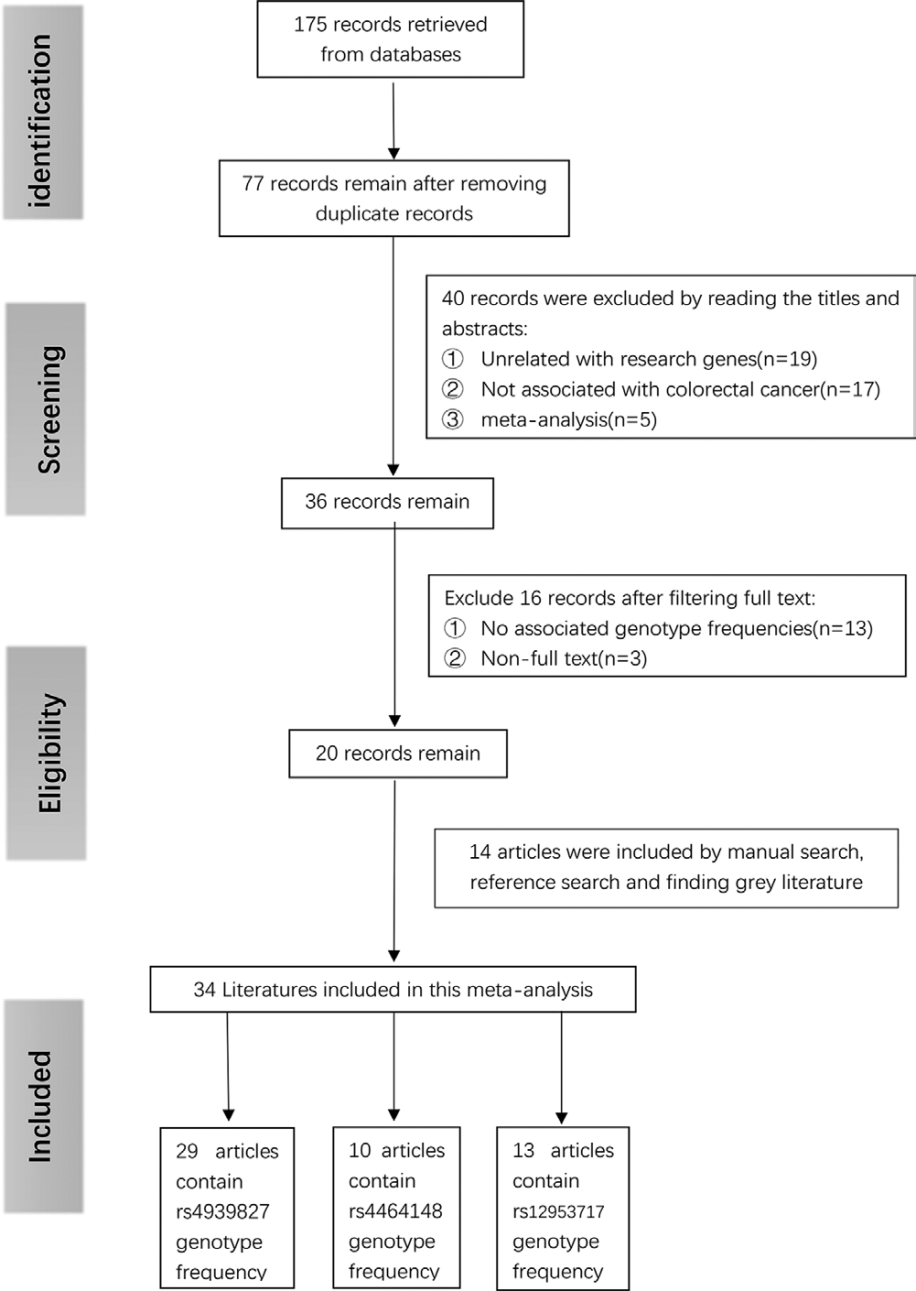
Flow chart of literature search and screening.

After searching methodically through 5 critical databases, we initially obtained 175 articles. After eliminating 98 repeated articles, 77 left. We then viewed the titles and abstracts and excluded 40 articles, including 19 non-related gene articles, 17 non-colorectal cancer-related articles, and 5 for meta-analysis. We read the remaining 36 papers in full text and excluded 16 articles, of which 3 were non-full-text articles, and another 13 had no relevant genotype frequencies in the text. We then included an additional 14 articles that matched the inclusion criteria by reference search in the remaining articles and other searches, which contains 1 gray literature. A totally of 34 articles were included in the final meta-analysis. After dropping studies that were not eligible for HWE (n = 10), a totally of 62 case–control studies were subsequently incorporated, 37 of which focused specifically on the connection between the rs4939827 and the chance of CRC,^[7,8,14–18,23–41]^ while the number of studies examining the connection between rs4464148 and rs12953717 and CRC risk were 10 and 15, respectively.^[7,23–27,29,30,38,39,41–44]^ The above process can be understood more intuitively from Figure 1.

### 3.2. Characteristics of included studies

As shown in Table 1, we extracted data from the 34 articles, including 80,281 cases and 92,970 controls. The tumors studied in the meta-analysis were limited to CRC, with 21 studies involving Caucasian populations and 10 studies involving Asian populations; there was also 1 study of blacks, and the remainder were mixed population studies. Among the comprehensive case–control studies, 10 studies are not in compliance with HWE (*P* < .05), and the results were more convincing after removing studies that did not comply with HWE than those that did not; therefore, we excluded the data of these studies. Quality among studies was evaluated via The Newcastle-Ottawa scale; studies all scored more significant than 6, indicating that the contained studies in this article were of high quality.

**Table 1:** The primary data information that is extracted from the incorporated articles.

SNPs	First Author	yr	Country	Ethnicity	Control source	Cancer type	Case	Control	HWE (P)	Genotyping method	NOS
***rs4939827***							***CC/TC/TT***	***CC/TC/TT***			
*(T > C*)	Broderick										
	-A group	2007	UK	Caucasian	NA	colon	153/449/328	229/480/251	0.987	Illumina	7
	-B group	2007	UK	Caucasian	NA	colon	852/2178/1392	845/1915/1084	0.989	Allele-PCR	7
	-C group	2007	UK	Caucasian	NA	colon	387/982/623	410/840/430	0.995	Allele-PCR	7
	-D group	2007	UK	Caucasian	NA	colon	194/477/292	76/171/96	0.993	Allele-PCR	7
	Tenesa										
	Scotland	2008	Scotland	Caucasian	NA	colon	538/1521/926	706/1508/845	0.506	Illumina	7
	-Japan	2008	Japan	Asian	NA	colon	233/1582/2576	131/1028/2019	0.992	TaqMan	7
	-Canada	2008	Canada	Caucasian	NA	colon	225/593/355	284/576/322	0.402	TaqMan	7
	-England	2008	England	Caucasian	NA	colon	418/1120/694	546/1126/578	0.959	TaqMan	7
	-Spain	2008	Spain	Caucasian	NA	colon	62/156/131	57/143/95	0.808	TaqMan	7
	-Scotland	2008	Scotland	Caucasian	NA	colon	156/420/254	189/446/288	0.497	TaqMan	7
	-Israel	2008	Israel	Caucasian	NA	colon	267/638/447	312/627/397	**0.035**	TaqMan	7
	-Germany	2008	Germany	Caucasian	NA	colon	420/1071/659	541/1057/530	0.762	TaqMan	7
	-Germany	2008	Germany	Caucasian	NA	colon	289/617/412	378/704/358	0.403	TaqMan	7
	Curtin	2009	Multi	Caucasian	PB	colon	221/520/324	229/538/274	0.251	SNPlex	8
	Thompson	2009	US	Mixed	PB	colon	125/275/154	146/378/185	0.064	TaqMan	7
	Pittman	2009	UK	Caucasian	HB	colon	785/1250/497	725/1300/582	0.987	Mixed	7
	Slattery	2010	US	Caucasian	Mixed	colon	360/773/457	492/992/503	0.947	TaqMan	7
	Xiong	2010	China	Asian	PB	colon	1370/677/77	1442/570/74	0.060	PCR-RFLP	8
	von Hoslt	2010	Sweden	Caucasian	HB	colon	395/886/501	387/884/408	**0.029**	TaqMan	8
	Kupfer	2010	US	African	HB	colon	379/340/76	455/429/101	0.993	MassARRAY	7
		2010	US	Caucasian	HB	colon	88/199/112	85/183/99	0.981	MassARRAY	7
	Mates	2010	Rome	Caucasian	PB	colon	28/37/27	15/57/23	**0.042**	Centaurus	6
	Mates	2011	Rome	Caucasian	PB	colon	42/69/42	32/106/43	**0.018**	Centaurus	6
	Cui	2011	Japan	Asian	PB	colon	1628/1007/155	2247/1190/147	0.501	Illumina	8
	Li	2011	China	Asian	PB	colon	73/53/12	81/73/14	0.665	MassARRAY	8
	Ho	2011	China	Asian	HB	colon	343/420/129	376/405/109	0.997	MassARRAY	7
	Song	2012	China	Asian	HB	colon	399/232/10	732/272/33	0.214	TaqMan	7
	Lubbe	2012	UK	Caucasian	HB	colon	444/969/624	1394/3021/1636	0.993	Allele-PCR	7
	Garcia-Albeniz	2012	US	Caucasian	HB	colon	90/233/118	538/1120/600	0.731	TaqMan	7
	Phipps	2012	US	Caucasian	HB	colon	657/1526/884	574/1597/1112	0.988	Illumina	7
	Kirac	2013	Croatia	Caucasian	PB	colon	63/143/96	172/291/131	0.705	TaqMan	8
	Yang	2014	China	Asian	PB	colon	342/298/65	891/752/159	0.985	MassARRAY	7
	Kurlapska	2014	Poland	Caucasian	PB	colon	54/93/65	716/1394/730	0.330	TaqMan	7
	Zhang	2014	MC	Asian	PB	colon	400/277/51	1894/1170/212	0.086	Mixed	7
	Hong	2015	Korea	Asian	PB	colon	126/63/9	182/127/19	0.608	Illumina	7
	Baert-Desurmont	2016	French	Caucasian	HB	colon	89/157/104	191/493/343	0.555	snapshot	7
	Abd EI-Fattah	2016	Egypt	Caucasian	NA	colon	20/35/22	11/15/10	0.319	TaqMan	7
	Alonso-Molero	2017	MC	Caucasian	PB	colon	176/524/387	495/1185/729	0.738	Illumina	7
	Shaker	2018	Egypt	Caucasian	HB	colon	13/44/29	13/15/8	0.367	Taq Man	7
	Reilly	2021	MC	Caucasian	HB	colon	9/16/5	9/37/14	0.061	Amplifluor	6
	Alidoust	2022	Iran	Caucasian	NA	colon	89/83/37	78/101/16	0.330	ARMS	7
***rs4464148***							***TT/TC/CC***	***TT/TC/CC***			
*(T > C*)	Broderick										
	-A group	2007	UK	Caucasian	NA	colon	389/425/116	486/394/80	0.991	Illumina	7
	-B group	2007	UK	Caucasian	NA	colon	2017/1952/472	1886/1617/346	0.982	Allele-PCR	7
	-C group	2007	UK	Caucasian	NA	colon	922/845/193	827/696/146	0.980	Allele-PCR	7
	-D group	2007	UK	Caucasian	NA	colon	422/408/99	171/137/27	0.952	Allele-PCR	7
	Thompson	2009	US	Mixed	PB	colon	269/231/61	342/324/53	**0.045**	TaqMan	7
	Curtin	2009	US	Caucasian	PB	colon	503/472/95	535/423/89	0.678	SNPlex	8
	Pittman	2009	UK	Caucasian	HB	colon	1161/1107/264	1095/1277/235	**<0.001**	Mixed	7
	Ho	2011	China	Asian	HB	colon	739/146/7	770/116/4	0.869	MassARRAY	7
	Zhang	2014	MC	Asian	PB	colon	1/52/675	14/305/2957	**0.045**	TaqMan	7
	Kurlapska	2014	Poland	Caucasian	PB	colon	1214/1228/400	84/96/33	0.523	TaqMan	7
	Damavand	2015	Iran	Caucasian	HB	colon	138/78/37	113/101/20	0.700	PCR-RFLP	7
	Serrano-fernadez	2015	MC	Caucasian	PB	colon	507/517/141	561/490/114	0.643	Taqman	8
	Reilly	2021	MC	Caucasian	HB	colon	10/16/2	27/23/5	0.974	Amplifluor	6
***rs12953717***							***CC/TC/TT***	***CC/TC/TT***			
*(C > T*)	Broderick										
	A group	2007	UK	Caucasian	NA	colon	159/309/151	326/467/167	0.991	Illumina	7
	B group	2007	UK	Caucasian	NA	colon	1247/2204/973	1248/1898/722	0.994	Allele-PCR	7
	C group	2007	UK	Caucasian	NA	colon	582/991/422	558/834/312	0.990	Allele-PCR	7
	D group	2007	UK	Caucasian	NA	colon	277/468/198	106/168/67	0.976	Allele-PCR	7
	Middeldorp	2009	Netherlands	Caucasian	HB	colon	301/493/201	482/643/215	0.982	KASPar	6
	Curtin	2009	US	Caucasian	PB	colon	314/530/226	332/521/188	0.509	SNPlex	8
	Thompson	2009	US	Mixed	PB	colon	196/248/116	220/370/129	0.218	TaqMan	7
	Pittman	2009	UK	Caucasian	HB	colon	716/1261/555	859/1275/473	0.998	Mixed	7
	Kupfer	2010	US	African	HB	colon	401/327/67	525/388/72	0.979	MassARRAY	7
		2010	US	Caucasian	HB	colon	197/121/81	119/180/68	0.996	MassARRAY	7
	Slattery	2010	US	Caucasian	Mixed	colon	503/754/332	676/928/327	0.779	Illumina	7
	Li	2011	China	Asian	PB	colon	57/79/6	90/63/13	0.672	MassARRAY	8
	Ho	2011	China	Asian	HB	colon	276/343/97	304/345/65	**0.018**	MassARRAY	7
	Scollen	2011	UK	Mixed	NA	colon	710/1031/425	730/1083/437	0.326	TaqMan	7
	Zhang	2014	China	Asian	PB	colon	418/263/47	1947/1135/194	0.096	TaqMan	7
	Damavand	2015	Iran	Caucasian	HB	colon	78/90/66	68/97/88	**<0.001**	PCR-RFLP	7
	Lu	2015	China	Asian	NA	colon	401/49/127	379/37/169	**<0.001**	PCR-RFLP	6
	Reilly	2021	MC	Caucasian	HB	colon	9/13/8	19/30/11	0.889	Amplifluor	6

Bold indicates that the result of HWE is less than 0.05 (n = 10).

HB = hospital based, HWE = Hardy-Weinberg equilibrium, MC = studies in the article were not conducted in the same country or region, mixed (control source) = the control group was derived from both PB and HB, Mixed (ethnicity) = Study subjects belong to two or more races that cannot be grouped together, Multi (country) = multi-center study, NA = the source of the control group was unclear, NOS = The Newcastle-Ottawa scale, PB = population based, SNPs = single nucleotide polymorphisms.

### 3.3. Meta-analysis findings

Table 2 shows the outcomes of meta-analysis for all snps.

**Table 2:** Summary of the correlation between SMAD7 polymorphisms and CRC risk in five models.

SNPs	Dominant model		Recessive model		Homozygous model		Heterozygous model		Additive model	
	OR (95% CI)	*P*/*I*^2^ (%)	OR (95% CI)	*P*/*I*^2^ (%)	OR (95% CI)	*P*/*I*^2^ (%)	OR (95% CI)	*P*/*I*^2^ (%)	OR (95% CI)	*P*/*I*^2^ (%)
**rs4939827 (T > C**)	**CC + CT vs TT**		**CC vs CT + TT**		**CC vs TT**		**CT vs TT**		**C vs T**	
Removel unHWE	**0.89 (0.83,0.97**)	**<0.01/80.2%**	**0.89 (0.83,0.96**)	**<0.01/78.4%**	**0.84 (0.76,0.93**)	**<0.01/82.6%**	**0.91 (0.85,0.97**)	**<0.01/68.3%**	**0.91 (0.87,0.96**)	**<0.01/84.7%**
Include unHWE	0.89 (0.77,1.04)	<0.01**/**95.5%	0.91 (0.85,0.97)	<0.01**/**78.1%	0.85 (0.77,0.94)	<0.01**/**81.2%	0.90 (0.85,0.96)	<0.01/66.6%	0.94 (0.88,0.99)	<0.01/88.4%
**Subgroup** #										
Ethnicity										
Caucasian	**0.87 (0.8,0.94**)	**<0.01/80.3%**	**0.87 (0.79,0.95**)	**<0.01/80.7%**	**0.79 (0.7,0.89**)	**<0.01/85.3%**	**0.88 (0.83,0.94**)	**<0.01/64.3%**	**0.89 (0.84,0.95**)	**<0.01/85.3%**
Asian	0.99 (0.82,1.2)	<0.01/71.0%	0.93 (0.82,1.04)	<0.01/73.2%	0.98 (0.79,1.21)	<0.01/68.2%	1.04 (0.88,1.23)	0.01/60.4%	0.95 (0.85,1.07)	<0.01/85.1%
African	1.08 (0.79,1.48)	–/–	1.06 (0.88,1.28)	–/–	1.11 (0.8,1.54)	–/–	1.05 (0.76,1.46)	–/–	1.05 (0.91,1.21)	–/–
Mixed	0.92 (0.71,1.48)	–/–	1.12 (0.86,1.47)	–/–	1.03 (0.75,1.42)	–/–	0.87 (0.67,1.14)	–/–	1.01 (0.86,1.18)	–/–
Control source										
PB	0.82 (0.76,0.89)	0.474/0.0%	**0.90 (0.82,0.99**)	**0.014/55.0%**	0.80 (0.71,0.92)	0.082/40.0%	0.85 (0.78,0.92)	0.672/0.0%	**0.9 (0.84,0.96**)	**0.016/54.1%**
HB	1.09 (0.94,1.27)	<0.01/77.5%	0.99 (0.85,1.15)	<0.01/82.3%	1.07 (0.88,1.31)	<0.01/81.6%	1.05 (0.92,1.19)	<0.01/65.8%	1.0 (0.9,1.1)	<0.01/84.1%
Mixed	0.77 (0.66,0.89)	–/–	0.89 (0.76,1.04)	–/–	0.81 (0.67,0.97)	–/–	0.86 (0.73,1.00)	–/–	0.89 (0.82,0.98)	–/–
NA	**0.83 (0.75,0.93**)	**<0.01/84.1%**	**0.81 (0.74,0.88**)	**<0.01/64.6%**	**0.73 (0.65,0.83**)	**<0.01/76.3%**	**0.87 (0.79,0.96**)	**<0.01/77.7%**	**0.87 (0.8,0.93**)	**<0.01/85.3%**
Sample scale										
LARGE	**0.90 (0.83, 0.97**)	**<0.01/82.8%**	**0.88 (0.82, 0.95**)	**<0.01/81.4%**	**0.84 (0.76, 0.93**)	**<0.01/86.9%**	**0.92 (0.86, 0.98**)	**<0.01/73.4%**	**0.91 (0.87, 0.96**)	**<0.01/86.4%**
SMALL	0.85 (0.62, 1.18)	<0.01/67.2%	0.96 (0.74, 1.23)	<0.01/60.8%	0.82 (0.67, 1.02)	<0.01/70.1%	0.89 (0.72, 1.1)	<0.01/50.3%	0.92 (0.7, 1.23)	0.09/50.2%
**rs4464148 (T > C**)	**CC + CT vs TT**		**CC vs CT + TT**		**CC vs TT**		**CT vs TT**		**C vs T**	
Removel unHWE	1.17 (1.07, 1.27)	0.058/45.3%	1.22 (1.11, 1.22)	0.404/3.9%	1.29 (1.17, 1.42)	0.286/17.1%	**1.13 (1.04, 1.24**)	**0.040/48.8%**	1.14 (1.09, 1.29)	0.100/38.7%
Include unHWE	1.11 (1.00, 1.23)	<0.01/72.1%	1.23 (1.14, 1.33)	0.492/0.0%	1.25 (1.15, 1.36)	0.220/22.1%	1.07 (0.95, 1.20)	<0.01/73.7%	**1.12 (1.05, 1.20**)	**<0.01/63.4%**
**Subgroup** #										
Ethnicity										
Caucasian	**1.15 (1.06, 1.26**)	**0.051/48.2%**	1.21 (1.11, 1.33)	0.34/11.4%	1.29 (1.17, 1.42)	0.229/24.2%	**1.12 (1.02, 1.23**)	**0.037/51.3%**	1.13 (1.09, 1.18)	0.102/39.9%
Asian	1.33 (1.02, 1.72)	–/–	1.75 (0.51, 6.01)	–/–	1.82 (0.53, 6.25)	–/–	1.31 (1.01, 1.71)	–/–	1.32 (1.03, 1.68)	–/–
Control source										
PB	1.11 (0.95, 1.31)	0.138/49.5%	1.11 (0.93, 1.32)	0.307/15.3%	1.17 (0.97, 1.41)	0.156/46.2%	1.12 (0.97, 1.29)	0.233/31.4%	**1.10 (1.01, 1.2**)	**0.098/56.9%**
HB	1.12 (0.72, 1.75)	0.039/69.3%	1.69 (1.03, 2.76)	0.639/0.0%	1.52 (0.91, 2.55)	0.894/0.0%	1.07 (0.59, 1.96)	<0.01/81.3%	1.17 (0.98, 1.4)	0.316/13.2%
NA	1.20 (1.09, 1.33)	0.134/46.3%	1.24 (1.11, 1.39)	0.361/6.4%	1.33 (1.18, 1.49)	0.162/41.6%	1.15 (1.07, 1.25)	0.316/15.2%	**1.14 (1.1, 1.19**)	**0.058/56.1%**
Sample scale										
LARGE	1.18 (1.10, 1.27)	0.151/34.8%	1.21 (1.10, 1.32)	0.419/1.3%	1.27 (1.15, 1.41)	0.12/40.7%	1.15 (1.09, 1.23)	0.379/6.3%	**1.14 (1.1, 1.19**)	**0.058/48.6%**
SMALL	1.03 (0.49, 2.18)	0.117/59.4%	1.67 (0.98, 2.87)	0.345/0.0%	1.48 (1.03, 2.11)	0.94/0.0%	0.99 (0.35, 2.84)	0.04/76.2%	1.03 (0.8, 1.33)	0.482/38.7%
**rs12953717 (C > T**)	**TT + TC vs CC**		**TT vs CC + TC**		**TT vs CC**		**TC vs CC**		**T vs C**	
Removel unHWE	**1.11 (1.01, 1.22**)	**<0.01/76.7%**	1.22 (1.15, 1.28)	0.209/22.0%	**1.25 (1.13, 1.38**)	**<0.01/55.3%**	1.06 (0.96, 1.18)	<0.01/77.2%	**1.11 (1.05, 1.17**)	**<0.01/67.0%**
Include unHWE	1.08 (0.98, 1.18)	<0.01/76.4%	**1.17 (1.07, 1.28**)	**<0.01/60.4%**	**1.18 (1.05, 1.33**)	**<0.01/70.9%**	1.06 (0.97, 1.17)	<0.01/73.3%	**1.08 (1.01, 1.15**)	**<0.01/75.7%**
**Subgroup** #										
Ethnicity										
Caucasian	**1.13 (1.01, 1.28**)	**<0.01/78.1%**	1.26 (1.19, 1.34)	0.620/0.0%	**1.34 (1.21, 1.48**)	**0.053/46.2%**	1.07 (0.94, 1.21)	<0.01/79.1%	**1.14 (1.071.22**)	**<0.01/66.8%**
Asian	1.32 (0.83, 2.11)	0.048/74.4%	1.00 (0.73, 1.37)	0.17/46.9%	1.08 (0.79, 1.49)	0.426/0.0%	1.4 (0.78, 2.53)	0.017/82.4%	1.10 (0.97, 1.24)	0.337/0.0%
African	1.12 (0.93, 1.35)	–/–	1.17 (0.83, 1.65)	–/–	1.22 (0.85, 1.74)	–/–	1.10 (0.91, 1.34)	–/–	1.1 (0.95, 1.28)	–/–
Mixed	0.92 (0.78, 1.10)	0.175/45.7%	1.05 (0.92, 1.20)	0.306/4.6%	1.0 (0.86, 1.16)	0.959/0.0%	0.88 (0.68, 1.13)	0.07/69.6%	0.99 (0.92, 1.07)	0.739/0.0%
Control source										
PB	1.09 (0.88, 1.35)	0.019/70.0%	1.16 (1.0, 1.34)	0.406/0.0%	1.14 (0.97, 1.35)	0.562/0.0%	1.08 (0.83, 1.41)	<0.01/78.5%	1.07 (0.99, 1.16)	0.363/6.1%
HB	1.0 (0.75, 1.34)	<0.01/88.8%	1.3 (1.17, 1.44)	0.698/0.0%	1.23 (0.97, 1.57)	0.026/63.7%	0.93 (0.67, 1.28)	<0.01/89.8%	1.07 (0.91, 1.25)	<0.01/81.0%
Mixed	1.16 (1.01, 1.34)	–/–	1.3 (1.09, 1.54)	–/–	1.36 (1.13, 1.65)	–/–	1.09 (0.94, 1.27)	–/–	1.16 (1.06, 1.28)	–/–
NA	**1.17 (1.04, 1.32**)	**0.015/67.8%**	**1.18 (1.10, 1.27**)	**0.048/58.3%**	**1.28 (1.07, 1.54**)	**<0.01/74.3%**	1.12 (1.02, 1.23)	0.132/43.5%	**1.13 (1.04, 1.24**)	**<0.01/75.2%**
Sample scale										
LARGE	**1.17 (1.10, 1.25**)	**0.073/42.8%**	1.21 (1.15, 1.28)	0.339/10.8%	**1.26 (1.13, 1.41**)	**0.046/55.7%**	**1.12 (1.07, 1.17**)	**<0.01/0.0%**	**1.11 (1.04, 1.17**)	**<0.01/71.2%**
SMALL	0.93 (0.63, 1.4)	<0.01/85.1%	1.42 (1.03, 1.97)	0.103/56.1%	1.21 (1.0, 1.47)	0.01/60.0%	0.96 (0.62, 1.47)	<0.01/89.7%	1.27 (0.94, 1.72)	0.888/0.0%

**Bold font** indicates statistically significant results.

CRC = colorectal cancer, HB = hospital based, HWE **=** Hardy-Weinberg equilibrium, LARGER = the total sample size in the study was greater than 1000, Mixed (control source) = the control group was derived from both PB and HB, Mixed (ethnicity) = Study subjects belong to two or more races that cannot be grouped together, NA **=** the source of the control group was unclear, PB = population based, SMAD7 = Sma-and Mad-Related Protein 7, SMALL = the total sample size in the study was less than 1000, unHWE = Not in accordance with the Hardy-Weinberg equilibrium study.

#, Data for subgroup analysis were obtained from the excluded unHWE study.

#### 3.3.1. RS4939827 (T > C).

**Figure 2.**
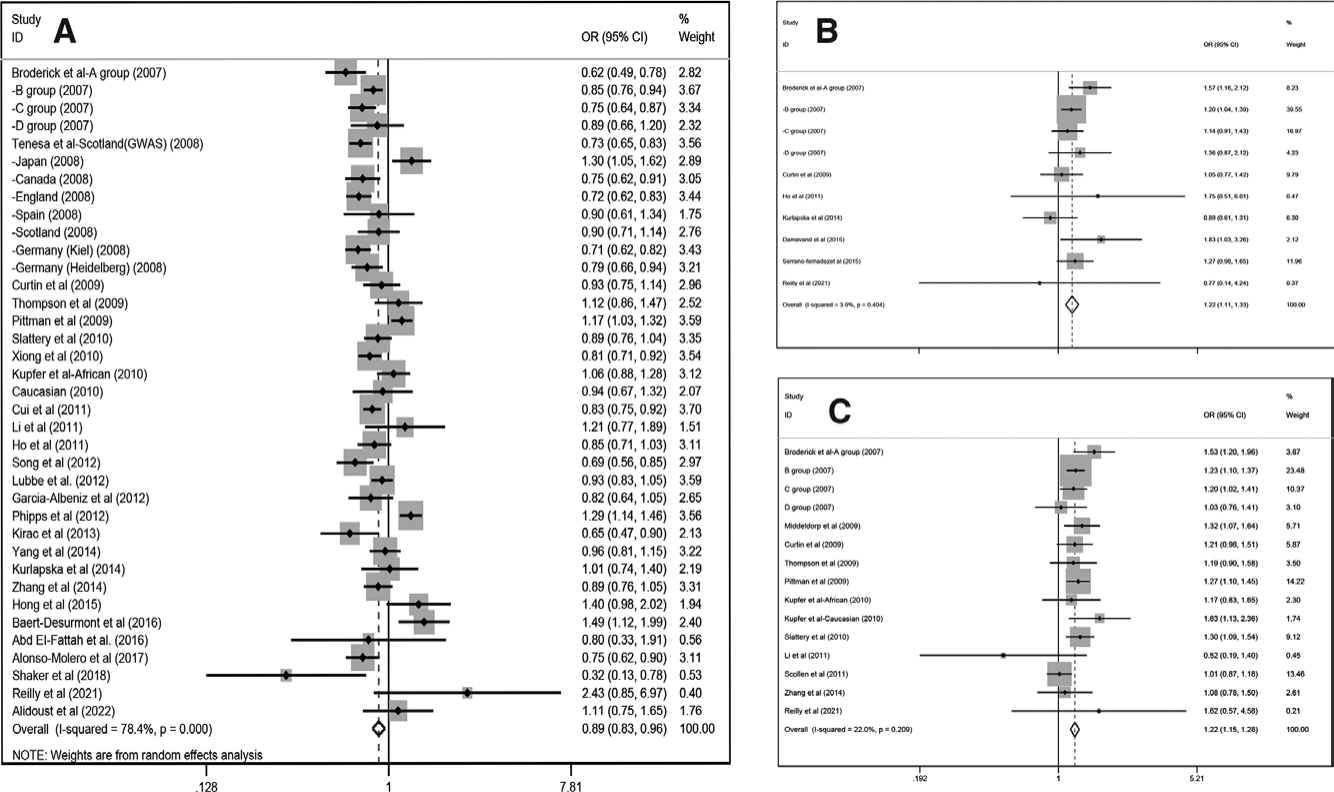
Forest plot of the recessive model for the correlation between three polymorphisms and CRC susceptibility. (A): Forest plot of the recessive model for rs4939827 polymorphism. (B): Forest plot of the recessive model for rs4464148 polymorphism. (C): Forest plot of the recessive model for rs12953717 polymorphism. CRC = colorectal cancer.

Our research uncovered that *C* allele of rs4939827 (46,784 cases, 60,938 controls) can reduced the overall CRC risk (dominant, OR/[95% CI] = 0.89/[0.83–0.97]; recessive, OR/[95% CI] = 0.89/[0.83–0.96]; homozygous, OR/[95% CI] = 0.84/[0.76–0.93]; heterozygous, OR/[95% CI] = 0.91/[0.85–0.97]; additive, OR/[95% CI] = 0.91/[0.87–0.96]). All these 5 types of models were analyzed using random effects model because of the significant heterogeneity (Fig. 2A). We in turn proceeded to subgroup analysis by ethnicity, control group source and sample, and the results are shown in Table 2. A detailed subgroup analysis of ethnicity unveiled that rs4939827 was only connected with Caucasians (dominant model, OR/[95% CI] = 0.87/[0.80–0.94]; recessive model, OR/[95% CI] = 0.87/[0.79–0.95]; homozygous model, OR/[95% CI] = 0.79/[0.70–0.89]; heterozygous model, OR/[95% CI] = 0.88/[0.83–0.94]; additive model, OR/[95% CI] = 0.89/[0.84–0.95]) and not with other ethnical groups. For the control source subgroup analysis, we found that this snp was a protective effect against CRC in the population-based groups (recessive model, OR/[95% CI] = 0.90/[0.82–0.99]; additive model, OR/[95% CI] = 0.90/[0.84–0.96]) and unknown source groups (dominant model, OR/[95% CI] = 0.89/[0.75–0.93]; recessive model, OR/[95% CI] = 0.81/[0.74–0.88]; homozygous model, OR/[95% CI] = 0.73/[0.65–0.83]; heterozygous model, OR/[95% CI] = 0.87/[0.79–0.96]; additive model, OR/[95% CI] = 0.87/[0.80–0.93]), but not in the hospital-based and mixed groups.

For the sample subgroup analysis, we found that the polymorphism reduced CRC risk in the large sample subgroup (dominant model, OR/[95% CI] = 0.90/[0.83–0.97]; recessive model, OR/[95% CI] = 0.88/[0.82–0.95]; homozygous model, OR/[95% CI] = 0.84/[0.76–0.93]; heterozygous model, OR/[95% CI] = 0.92/[0.86–0.98]; additive model, OR/[95% CI] = 0.91/[0.87–0.96]) compared to the small sample subgroup.

#### 3.3.2. RS4464148 (T > C).

In this part, random effect model was utilized for the dominant and heterozygous model and fixed-effect models for the recessive, homozygous and additive model, and then the results significantly indicated that rs4464148 (14,510 cases,10,417controls) increased the CRC risk (heterozygous, OR/[95% CI] = 1.12/[1.02-1.23], Fig. 2B). We analyzed this polymorphism in 3 subgroups and drew relevant conclusions that allelic mutations from *C* to *T* can increase CRC risk in Caucasian populations (dominant, OR/[95% CI] = 1.15/[1.06–1.26]; heterozygous, OR/[95% CI] = 1.12/[1.02–1.23]) but have no impact on Asian races. For control group source subgroup analysis, In the unknown origin group, it also revealed a relationship among rs4464148 and CRC (additive, OR/[95% CI] = 1.14/[1.10–1.19]), The correlation between the snp and CRC was also suggested in the population-based group (additive model, OR/[95% CI] = 1.10/[1.01–1.20]). And the large sample group also suggested a remarkable connection between this gene and CRC (additive model, OR/[95% CI] = 1.14/[1.10–1.19]), all outcomes of subgroup are shown in Table 2.

#### 3.3.3. RS12953717 (C > T).

As for this snp, After collecting and processing the data, we can drag a conclusion that rs12953717 (18,987 cases, 21,615 controls) polymorphism increased risk of CRC (recessive, OR/[95% CI] = 1.22/[1.15–1.28]; homozygous, OR/[95% CI] = 1.25/[1.13–1.38]; additive, OR/[95% CI] = 1.11/[1.05–1.17], Fig. 2C),it indicates that *C*-allele to *T*-allele mutations may cause CRC, In further subgroup analyses, this gene polymorphism was statistically related with CRC only in Caucasian populations (dominant, OR/[95% CI] = 1.13/[1.01–1.28]; homozygous, OR/[95% CI] = 1.34/[1.21–1.48]; additive, OR/[95% CI] = 1.14/[1.07–1.22]), for the control source subgroup analysis, we found that unknown source group(dominant, OR/[95% CI] = 1.17/[1.04–1.32]; recessive, OR/[95% CI] = 1.18/[1.10–1.27]; homozygous, OR/[95% CI] = 1.28/[1.07–1.54]; additive, OR/[95% CI] = 1.13/[1.04–1.24]) are related to CRC. The same results can also be derived from the sample subgroup analysis that rs12953717 was regarded as important contributing factors in LARGE group (dominant, OR/[95% CI] = 1.17/[1.10–1.25]; homozygous, OR/[95% CI] = 1.26/[1.13–1.41]; heterozygous, OR/[95% CI] = 1.12/[1.07–1.17]; additive, OR/[95% CI] = 1.11/[1.04–1.17]), while no statistical meaningfulness was found in the SMALL group.

#### 3.3.4. Heterogeneity analysis.

In data integration and analysis, we found that rs4939827 was highly heterogeneous (P_h_ < 0.01), for which we employed random effects model to pool ORs and perform subgroup analysis, this model is also used in dominant (I-squared = 45.3%, P_h_ = 0.058) and heterozygous model (I-squared = 48.8%, P_h_ = 0.040) of rs4464148 and rs12953717 for dominant (I-squared = 76.7%, P_h_ = 0.01), homozygous (I-squared = 55.3%, P_h_ = 0.005), heterozygous (I-squared = 77.2%, P_h_ = 0.01) and additive models (I-squared = 67.0%, P_h_ = 0.01), Instead, due to low heterogeneity, we applied fixed-effects models to subgroup analyses of the recessive (I-squared = 3.9%, P_h_ = 0.404), homozygous (I-squared = 17.1%, P_h_ = 0.286) and additive models (I-squared = 38.7%, P_h_ = 0.100) in rs4464148 and the recessive model (I-squared = 22.0%, P_h_ = 0.209) in rs12953717. Since the number of studies on these 3 polymorphisms belonging to SMAD7 exceeded 10, we used meta-regression to probe the root of their heterogeneity, then we found that the heterogeneity of rs12953717 was mainly derived from ethnicity (See Table S3, Supplemental Digital Content, http://links.lww.com/MD/I299, which show the results of meta-regression analysis of 3 polymorphisms). Although we did not identify any significant sources of heterogeneity in the meta-regressions about rs4939827 and rs4464148, the overall high heterogeneity should not be ignored, so subsequently subgroup analysis by ethnicity, control group source and sample can reveal that the heterogeneity of rs4464148 polymorphism mainly originates from the Caucasian group and hospital-based group of control group source, all subgroups of rs4939827 showed highly heterogeneous.

#### 3.3.5. Publication bias.

**Figure 3.**
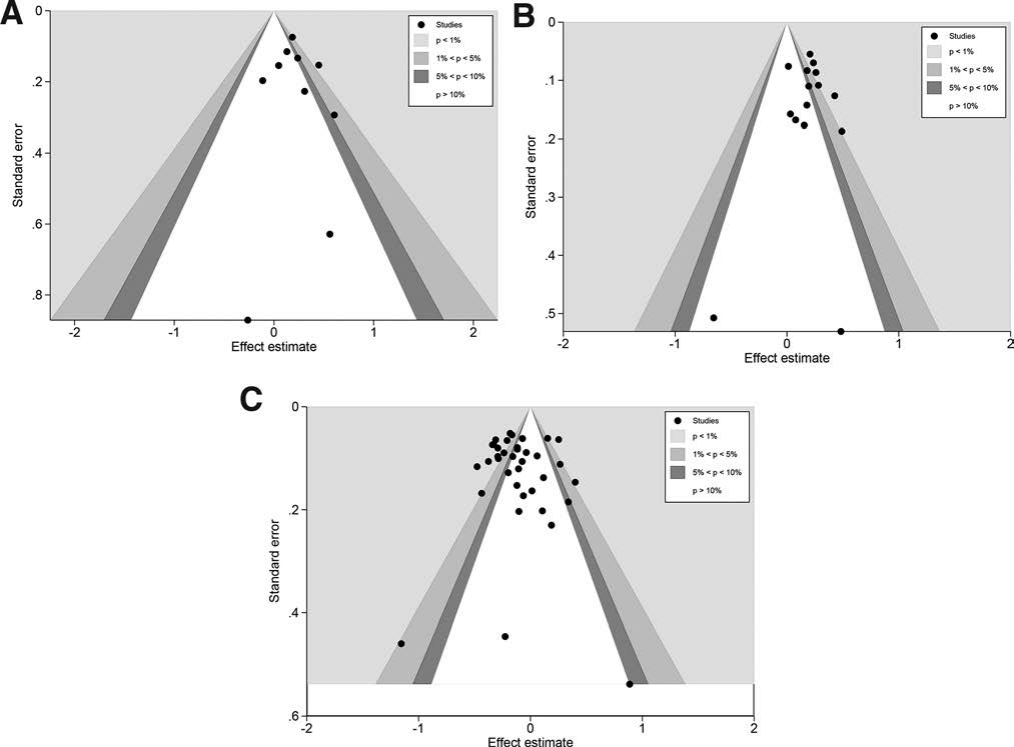
Contour-enhanced Funnel plot of the recessive model for three polymorphisms. (A): contour-enhanced Funnel plot of the recessive model for rs4464148. (B): contour-enhanced Funnel plot of the recessive model for rs12953717. (C): contour-enhanced Funnel plot of the recessive model for rs4939827.

The publication bias of the 5 models of rs4939827, rs4464148, and rs12953717 was evaluated separately using contour-enhanced funnel plots (Fig. 3), and we can detect noticeable dissymmetry in the funnel plots of dominant, recessive, homozygous, heterozygous, and additive models. However, this asymmetry was due to the distribution of studies in statistically significant regions outside the white of the funnel plot. In our further quantitative analysis of publication bias using Begg test and Egger test, no apparent publication bias was found in each of the 5 models (See Table S4, Supplemental Digital Content, http://links.lww.com/MD/I300, which show the results of publication bias) for these 3 gene polymorphisms, suggesting that the asymmetry in the funnel plot is caused by factors other than publication bias and most likely is caused by heterogeneity.

#### 3.3.6. Sensitivity analysis.

**Figure 4.**
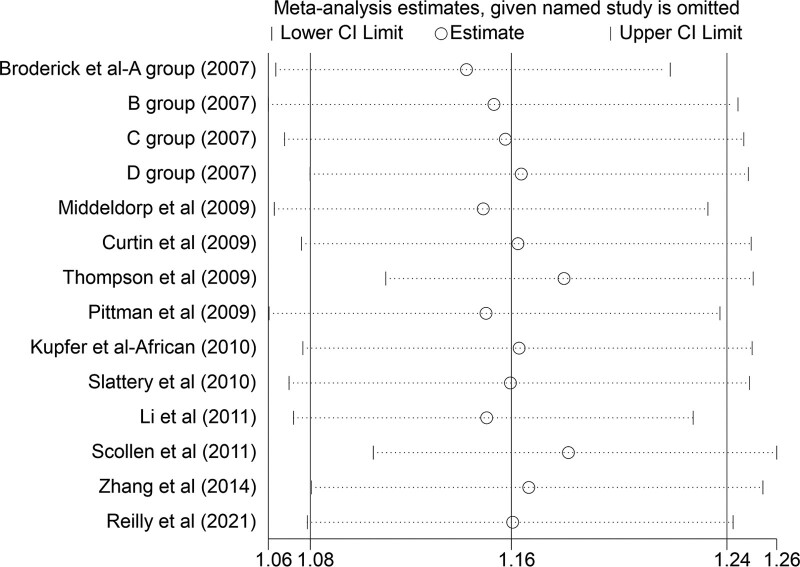
Results of sensitivity analysis after excluding one study of the dominant model in rs12953717.

We performed sensitivity analyses on the pooled results to assess the individual impact of each study. Removing any of the case–control studies did not result in a change in outcome except for the dominant model in rs12953717, where the results changed after excluding the 1 case–control study, which is the Caucasian group of Kupfer et al (399 cases,367controls). It suggests that this study had a distinctive effect on the results, making the results destabilizing (Fig. 4).

### 3.4. Result of FPRP analysis and TSA

**Figure 5.**
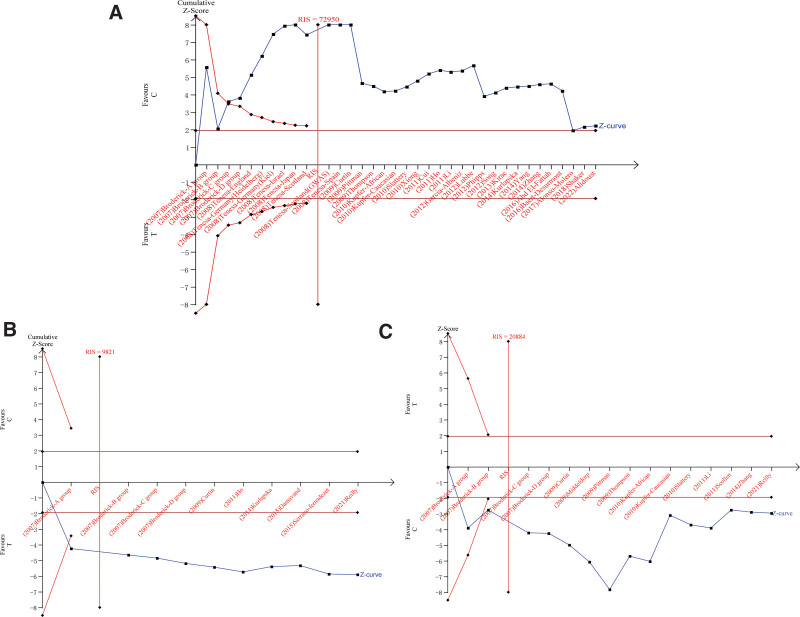
Results of sample evaluation of three polymorphisms in allelic model. (A) rs4939827; (B) rs4464148; (C) rs12953717.

The results of the false-positive report probability analysis are depicted in Table 3, which contains FPRPs for 3 polymorphisms with statistically significant ORs, all of which are less than 0.2 with an a priori probability of 0.1. It indicates that our results have a low probability of false positives and that our findings are noteworthy. We perform a trial sequential analysis for the allele model, and Figure 5 visualizes the results. We found that the *Z* curves for all 3 polymorphisms crossed the boundary curves, with cumulative *Z* values exceeding 1.96 (a = 0.05), and that the sample size exceeded the required information size. These results represent that our included sample size fulfills the sample size we need to draw factual conclusions, proving the reliability of our conclusions.

**Table 3:** Result of False-positive probability analysis at six prior probability levels.

Model	Subgroup	*P* value	OR (95% CI)	Statistical Power†	Prior probability					
					0.25	0.1	0.01	0.001	0.0001	0.00001
Rs4939827										
CC + CT vs TT	Remove HWE	.008	0.89 (0.83, 0.97)	1.000	0.023	0.067	0.441	0.888	0.988	0.999
	Caucasian	<.001	0.87 (0.8, 0.94)	1.000	0.001	0.004	0.040	0.296	0.808	0.977
	NA	.001	0.83 (0.75, 0.93)	1.000	0.004	0.012	0.116	0.570	0.930	0.993
	LAGER	<.001	0.90 (0.83, 0.97)	1.000	<0.001	<0.001	<0.001	<0.001	<0.001	<0.001
CC vs CT + TT	Remove HWE	.003	0.89 (0.83, 0.96)	1.000	0.008	0.022	0.202	0.718	0.962	0.996
	Caucasian	.002	0.87 (0.79, 0.95)	1.000	0.006	0.017	0.159	0.657	0.950	0.995
	PB	.03	0.90 (0.82, 0.99)	1.000	0.083	0.014	0.750	0.968	0.997	1.000
	NA	<.001	0.81 (0.74, 0.88)	1.000	<0.001	<0.001	<0.001	0.001	0.006	0.059
	LAGER	.001	0.88 (0.82, 0.95)	1.000	0.003	0.009	0.095	0.515	0.914	0.991
CC vs TT	Remove HWE	<.001	0.84 (0.76, 0.93)	1.000	0.002	0.007	0.072	0.440	0.887	0.987
	Caucasian	<.001	0.79 (0.7, 0.89)	0.997	<0.001	0.001	0.010	0.096	0.515	0.914
	PB	.002	0.80 (0.71, 0.92)	0.995	0.005	0.016	0.148	0.638	0.946	0.994
	NA	<.001	0.73 (0.65, 0.83)	0.917	<0.001	<0.001	<0.001	0.002	0.017	0.145
	LAGER	<.001	0.84 (0.76, 0.93)	1.000	0.002	0.007	0.072	0.440	0.887	0.987
CT vs TT	Remove HWE	.004	0.91 (0.85, 0.97)	1.000	0.011	0.033	0.273	0.791	0.974	0.997
	Caucasian	<.001	0.88 (0.83, 0.94)	1.000	<0.001	0.001	0.014	0.127	0.593	0.936
	NA	.006	0.87 (0.79, 0.96)	1.000	0.016	0.048	0.355	0.847	0.982	0.998
	LAGER	.01	0.92 (0.86, 0.98)	1.000	0.028	0.080	0.490	0.906	0.990	0.999
C vs T	Remove HWE	<.001	0.91 (0.87, 0.96)	1.000	0.002	0.005	0.052	0.354	0.846	0.982
	Caucasian	<.001	0.89 (0.84, 0.95)	1.000	0.001	0.004	0.044	0.317	0.823	0.979
	PB	.001	0.9 (0.84, 0.96)	1.000	0.004	0.012	0.120	0.579	0.932	0.993
	NA	<.001	0.87 (0.8, 0.93)	1.000	<0.001	<0.001	0.004	0.041	0.299	0.810
	LAGER	<.001	0.91 (0.87, 0.96)	1.000	0.002	0.005	0.052	0.354	0.846	0.982
Rs4464148*										
CC + CT vs TT	Caucasian	.003	1.15 (1.06, 1.26)	1.000	0.008	0.024	0.212	0.730	0.964	0.996
CT vs TT	Remove HWE	.01	1.13 (1.04, 1.24)	1.000	0.029	0.082	0.495	0.908	0.990	0.999
	Caucasian	.018	1.12 (1.02, 1.23)	1.000	0.051	0.138	0.637	0.947	0.994	0.999
C vs T	Include HWE	.001	1.12 (1.05, 1.20)	1.000	0.004	0.011	0.113	0.562	0.928	0.992
	PB	.03	1.10 (1.01, 1.2)	1.000	0.087	0.023	0.759	0.969	0.997	1.000
	NA	<.001	1.14 (1.1, 1.19)	1.000	<0.001	<0.001	<0.001	<0.001	<0.001	<0.001
	LAGER	<.001	1.14 (1.1, 1.19)	1.000	<0.001	<0.001	<0.001	<0.001	<0.001	<0.001
Rs12953717										
TT + TC vs CC	Remove HWE	.03	1.11 (1.01, 1.22)	1.000	0.084	0.015	0.751	0.968	0.997	1.000
	Caucasian	.05	1.13 (1.01, 1.28)	1.000	0.141	0.030	0.844	0.982	0.998	1.000
	NA	.01	1.17 (1.04, 1.32)	1.000	0.031	0.088	0.515	0.915	0.991	0.999
	LAGER	<.001	1.17 (1.10, 1.25)	1.000	<0.001	<0.001	<0.001	0.003	0.032	0.247
TT vs CC + TC	Include HWE	<.001	1.17 (1.07, 1.28)	1.000	0.002	0.006	0.057	0.381	0.860	0.984
	NA	<.001	1.18 (1.10, 1.27)	1.000	<0.001	0.001	0.001	0.010	0.092	0.504
TT vs CC	Remove HWE	<.001	1.25 (1.13, 1.38)	1.000	<0.001	<0.001	0.001	0.010	0.090	0.496
	Include HWE	.007	1.18 (1.05, 1.33)	1.000	0.020	0.057	0.399	0.870	0.985	0.999
	Caucasian	<.001	1.34 (1.21, 1.48)	0.987	<0.001	<0.001	<0.001	<0.001	<0.001	<0.001
	NA	.009	1.28 (1.07, 1.54)	0.954	0.027	0.077	0.480	0.903	0.989	0.999
	LAGER	<.001	1.26 (1.13, 1.41)	0.999	<0.001	0.001	0.006	0.053	0.361	0.850
TC vs CC	LAGER	<.001	1.12 (1.07, 1.17)	1.000	<0.001	<0.001	<0.001	<0.001	0.004	0.035
T vs C	Remove HWE	<.001	1.11 (1.05, 1.17)	1.000	<0.001	0.001	0.010	0.093	0.505	0.911
	Include HWE	.016	1.08 (1.01, 1.15)	1.000	0.047	0.128	0.618	0.942	0.994	0.999
	Caucasian	<.001	1.14 (1.07, 1.22)	1.000	<0.001	0.001	0.015	0.132	0.604	0.939
	NA	.01	1.13 (1.04, 1.24)	1.000	0.029	0.082	0.495	0.908	0.990	0.999
	LAGER	<.001	1.11 (1.04, 1.17)	1.000	<0.001	0.001	0.010	0.093	0.505	0.911

CI = confidence interval, GC = gastric cancer, HWE = Hardy-Weinberg equilibrium, LAGER = the total sample size in the study was greater than 1000, NA = control source unknow, OR = odds ratio, PB = population based.

* No statistically significant ORs for the recessive and Homozygous models in RS4464148.

† The statistical power is measured at an odd ratio of 1.5.

### 3.5. Outcome of bioinformation analysis

#### 3.5.1. Protein interactions network analysis.

**Figure 6.**
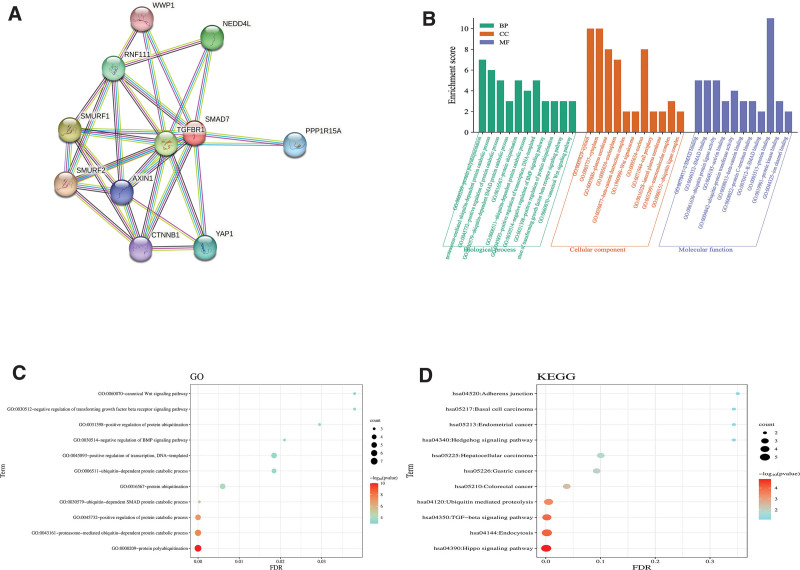
Gene relationship network diagram and enrichment analysis results of SMAD7. (A) Gene relationship network diagram of SMAD7. (B) Enrichment of SMAD7-related genes in each gene ontology (GO) term and gene counts. (C) The findings of gene ontology analysis. (D) The findings of Kyoto encyclopedia of genes and genomes analysis. FDR = false discovery rate, GO = gene ontology, KEGG = Kyoto encyclopedia of genes and genomes, SMAD7 = Sma-and mad-related protein 7.

We imaged the SMAD7 gene co-expression network based on the STRING database (Fig. 6A), which displayed us the count and names of genes with strong expression association with SMAD7. These include WWP1, NEDD4L, RNF111, SMURF1, TGFBR1, PPP1R15A, SMURF2, AXIN1, CTNNB1, and YAP1, with a total number of 11.

#### 3.5.2. Enrichment analysis.

Figure 6B shows the enrichment score of biological processes, cellular component and molecular function. Enrichment analysis with DAVID yielded 79 gene ontology terms and 11 Kyoto encyclopedia of genes and genomes terms, excluding those with false discovery rate < 0.05, leaving 20 gene ontology terms and 5 Kyoto encyclopedia of genes and genomes terms The bubble size was determined by the number of differential genes contained in the pathway, and the color represents the *P*-value, the smaller the *P*-value, the closer to red (Fig. 6C, D).

## 4. Discussion

SNPs are polymorphisms in DNA sequences caused by discrepancies in 1 base-pairs and such discrepancies include deletions, insertions, and substitutions. Some SNPs located within genes can directly alter protein structure and expression levels.^[45]^ Now that polymorphisms have been intensively studied, researchers have been constantly probing the molecular mechanisms underlying the association of polymorphisms with diseases. Much evidence suggests that they alter an individual’s genetic susceptibility to cancer by regulating gene expression.^[46]^ Mutations are irreversible variants in DNA that essentially include spontaneous or non-spontaneous mutations in the human genome. Most mutations are disease-causing in nature. Since mutations are influenced by both environment and genetics, the distribution of snp and mutations is region-specific and race-specific.^[46,47]^ SMAD7, as an inhibitor of the TGF-*β* signaling pathway, blocks TGF-*β* signaling through a negative feedback loop, and due to this inhibitory function, SMAD7 can antagonize a variety of TGF-beta-regulated cellular metabolic processes, for instance, cell proliferation, cell differentiation, apoptosis, adhesion, and migration, then impacting CRC progression.^[3,5,48]^ By including the maximum number of case–control studies to date, this analysis also consistently concluded that both the *T* allele of rs12953717 and the C allele of rs4464148 increased the risk of CRC whereas high expression of the C allele of rs4939827 reduced the risk of CRC.

A meta-analysis was written by Huang et al in 2016 also came to the non-contradictory conclusion,^[49]^ This article also included substantial case–control studies. However, the analysis of rs4939827 focused on the major gene (*T* allele) and had problems with the calculation of the HWE, leading to the inclusion of some case–control studies that did not conform to the HWE. In addition, that article did not conduct meta-regression analysis and subgroup analysis to explore the sources of heterogeneity further. Our researchers were fully aware of these shortcomings. The present meta-analysis included as many articles that met the criteria as possible while excluding case–control studies that did not conform to the HWE, which can be said to be the most relevant study with the highest number of cases so far and included 5 more case–control studies than the previous 1 to increase the accuracy of the results. We also reduced selection bias and improved the accuracy of results by searching in Chinese databases and adding gray literature.

Moreover, we found that all of the 3 polymorphisms had heterogeneity in this analysis, so we employed a meta-regression analysis with subgroup analysis by ethnicity, control group source, and sample to find the specific sources of heterogeneity. We found that the heterogeneity of rs12953717 and rs4464148 was principally derived from ethnicity. We further discovered that heterogeneity existed mainly in studies on Caucasian populations; the reasons may be related to the socio-economic situation, food habits, and the wide distribution of ethnic groups, the source of heterogeneity of rs4939827 may be multifaceted, such as the genotyping method of case–control studies, the source of subject recruitment, environmental factors, and dietary habits.^[50,51]^ The asymmetry of contour-enhanced funnel plots for the dominant, recessive, homozygous, heterozygous, and additive models may be associated with heterogeneity, and other results of Begg and Egger test did not find publication bias. For sensitivity analysis, we excluded each of the included studies 1 by 1 before combining effect sizes, and compared the new combined results with the results before the exclusion, and found no significant differences between the results of rs4464148 and rs4939827, which indicates that the results of the analysis of rs4464148 and rs4939827 in this article are robust and credible. In rs12953717, we found that the results changed after removing the Caucasian group of studies in the Kupfer et al study, which suggests that the results of the analysis with the removal of this study would be more credible.

It is worth noting that this meta-analysis also has inadequacies. For instance, we have only 1 study on the African population, and more studies may be needed in the future. The tumor we studied were also constrained to CRC; more research is needed in the future to explore the correlation between SMAD7 polymorphisms and cancer susceptibility further. Although smad7 has been extensively probed, the molecular mechanism of SMAD7 is not fully defined, so it cannot serve the clinic better. We also detected many co-expressed genes through bioinformatic analysis. We can further investigate the role of intracellular signaling pathways and gene co-expression, regarding the elucidation of the role of TGF-*β*1 signaling in specific pathogenic environments as an important direction for future research; this may pave the way for the development of strategies to modulate these disease processes to develop the most effective therapy plan better for CRC patients.^[52]^

## 5. Conclusion

To sum up the above, our study is very relevant. The results of our meta-analysis certified the noticeable relationship of the rs4939827 (T > C) variant with reduced CRC risk. However, the rs4464148 (T > C) and rs12953717 (C > T) variants were significantly correlated with an increased risk of CRC.

## Acknowledgements

This study was supported by grants from the General Science and Technology Program of the Health Commission of Jiangxi Province (202210415).

## Author contributions

**Conceptualization:** Qiang Xiao, Guomin Zhu.

**Data curation:** Jian Chen, Shukun Zeng.

**Formal analysis:** Qiang Xiao, Jian Chen, Hu Cai.

**Funding acquisition:** Guomin Zhu.

**Resources:** Qiang Xiao, Shukun Zeng, Hu Cai.

**Software:** Jian Chen.

**Visualization:** Qiang Xiao.

**Writing – original draft:** Qiang Xiao, Shukun Zeng.

**Writing – review & editing:** Jian Chen, Jia Zhu, Hu Cai.

## Supplementary Material


