# Supplementary material for: Association of several loci of SMAD7 with colorectal cancer: A meta-analysis based on case–control studies
Source: Medicine (Baltimore). 2023 Jan 6;102(1):e32631. doi: 10.1097/MD.0000000000032631 (PMC9829263; doi:10.1097/MD.0000000000032631)
Supplement: Supplementary file 1 [file medi-102-e32631-s001.pdf]

Supplemental Table 1: Literature Searching Strategy.

Database	Search Strategy
PubMed	((((((Case-Control Study[MeSH Terms]) OR (Case-Control Studies[Title/Abstract])) OR (Studies, Case-Control[Title/Abstract])) OR (Study, Case-Control[Title/Abstract])) AND ((((((((Single Nucleotide Polymorphism[MeSH Terms])) OR (Nucleotide Polymorphism, Single[Title/Abstract])) OR (Nucleotide Polymorphisms, Single[Title/Abstract])) OR (Polymorphisms, Single Nucleotide[Title/Abstract]) OR (Single Nucleotide Polymorphisms[Title/Abstract])) OR (SNPs[Title/Abstract])) OR (Polymorphism, Single Nucleotide[Title/Abstract])))) AND (((((smad7 protein[MeSH Terms]) OR (madh7 protein[MeSH Terms])) OR (rs4939827[Title/Abstract])) OR (rs12953717[Title/Abstract])) OR (rs4464148[Title/Abstract]))) AND (((((((((((((Neoplasia[MeSH Terms])) OR (Tumor[Title/Abstract])) OR (Neoplasm[Title/Abstract])) OR (Tumors[Title/Abstract])) OR (Neoplasias[Title/Abstract])) OR (Cancer[Title/Abstract])) OR (Malignant Neoplasm[Title/Abstract])) OR (Malignancy[Title/Abstract])) OR (Malignancies[Title/Abstract])) OR (Malignant Neoplasms[Title/Abstract])) OR (Neoplasm, Malignant[Title/Abstract])) OR (Neoplasms, Malignant[Title/Abstract])))
Web of Science	(((TS=("Case-Control Stud*")) OR TS=("Stud*, Case-Control")) OR TS=("Case-Comparison Stud*")) and ((((((((TS=("Single Nucleotide Polymorphism*")) OR TS=("Nucleotide Polymorphism*, Single")) OR TS=("Polymorphism*, Single Nucleotide")) OR TS=(Allelomorph*)) OR TS=(SNPs)) OR TS=(Allele)) OR TS=(Genetic Mutation)) OR TS=(Mutation*)) and (((((((((TS=(Smad7 Protein)) OR TS=(Madh7 Protein)) OR TS=(Smad7)) OR TS=(Madh7)) OR TS=(Sma- and Mad-Related)) OR TS=(Mothers Against Decapentaplegic Homolog)) OR AB=(rs12953717)) OR AB=(rs4939827)) OR AB=(rs4464148)) and ((((((TS=(Neoplas*)) OR TS=(Cancer*)) OR TS=(Tumor*)) OR TS=(Malignanc*)) OR TS=("Malignant Neoplasm*")) OR TS=("Neoplasm*, Malignant"))
Embase	('malignant neoplasm'/exp OR 'cancer':ti,ab,kw OR 'cancers':ti,ab,kw OR 'malignant neoplasia':ti,ab,kw OR 'malignant neoplasm':ti,ab,kw OR 'malignant neoplastic disease':ti,ab,kw OR 'malignant tumor':ti,ab,kw OR 'malignant tumour':ti,ab,kw OR

	'neoplasia, malignant':ti,ab,kw OR 'tumor, malignant':ti,ab,kw OR 'tumour, malignant':ti,ab,kw) and ('case control study'/exp OR 'case control study' OR 'case-control studies' OR 'case-control study' OR 'control study, case' OR 'matched case control' OR 'matched case control studies' OR 'matched case control study') and ('smad7 protein'/exp OR 'sma and mad related protein 7':ti,ab,kw OR 'smad7 protein':ti,ab,kw OR 'smad7 transcription factor':ti,ab,kw OR 'protein smad7':ti,ab,kw OR 'transcription factor smad7':ti,ab,kw OR rs4939827:ti,ab OR rs12953717:ti,ab OR rs4464148:ti,ab) and ('single nucleotide polymorphism'/exp OR 'polymorphism, single nucleotide':ti,ab,kw OR 'single nucleotide polymorphism':ti,ab,kw OR 'single nucleotide variant':ti,ab,kw OR 'single nucleotide variation':ti,ab,kw)
China National Knowledge Infrastructure (CNKI)	(（主题：肿瘤）OR（主题：癌症）OR（主题：恶性肿瘤）OR（主题：癌）) AND (（主题：Smad 7）OR（主题：sma and mad related protein 7）OR（主题：rs4939827）OR（主题：rs4464148）OR（主题：rs12953717）) AND (（主题：单核苷酸）OR（主题：多态性）OR （主题：单核苷酸多肽现象） OR（主题：SNP）OR（主题：Polymorphism, Single Nucleotide）OR（主题：Polymorphism）OR（主题：突变）OR（主题：等位基因）) AND (（摘要：病例对照研究）OR（摘要：基于病例研究）OR（摘要：病例比较研究）OR（摘要：病例关联研究） OR（摘要：配对病例对照研究）OR（摘要：成批病例对照研究）)
Wan Fang database	（主题：（＂肿瘤＂）OR 主题：(＂癌症＂)OR 主题：（＂恶性肿瘤＂）OR 主题：（＂癌＂）） AND （主题：（＂Smad 7＂）OR 主题：（＂sma and mad related protein 7＂）OR 主题：（＂rs4939827＂）OR 主题：（＂rs4464148＂）OR 主题：（＂rs12953717＂）） AND （主题：（＂单核苷酸＂）OR 主题：（＂多态性＂）OR 主题：（＂单核苷酸多肽现象＂） OR 主题：（＂SNP＂）OR 主题：（＂Polymorphism, Single Nucleotide＂）OR 主题：（＂Polymorphism＂）OR 主题：（＂突变＂）OR 主题：（＂等位基因＂）） AND （摘要：（＂病例对照研究＂）OR 摘要：（＂基于病例研究＂）OR 摘要：（＂病例比较研究＂）OR 摘要：（＂病例关联研究＂） OR 摘要：（＂配对病例对照研究＂）OR 摘要：（＂成批病例对照研究＂））
