# Supplementary material for: Association of several loci of SMAD7 with colorectal cancer: A meta-analysis based on case–control studies
Source: Medicine (Baltimore). 2023 Jan 6;102(1):e32631. doi: 10.1097/MD.0000000000032631 (PMC9829263; doi:10.1097/MD.0000000000032631)
Supplement: Supplementary file 2 [file medi-102-e32631-s002.pdf]

Supplemental Table 2. Assessing the quality of incorporated studies using the Newcastle-Ottawa scale.

author	year	Selection				Comparability	Exposure			Quality	Score
RS4939827		Item 1	Item 2	Item 3	Item 4	Item 5	Item 6	Item 7	Item 8		
Broderick	2007	*	*		*	**	*	*		High	7
Tenesa	2008	*	*		*	**	*	*		High	7
Curtin	2009	*	*	*	*	**	*	*		High	8
Thompson	2009	*	*	*	*	**		*		High	7
Pittman	2009	*	*		*	**	*	*		High	7
Slattery	2010	*	*		*	**	*	*		High	7
Xiong	2010	*	*	*	*	**	*	*		High	8
von Hoslt	2010	*	*		*	**	*	*		High	7
Kupfer	2010	*	*		*	**	*	*		High	7
Mates	2010	*	*	*		**		*		High	6
Mates	2011	*	*	*		**		*		High	6
Cui	2011	*	*	*	*	**	*	*		High	8
Li	2011	*	*	*	*	**	*	*		High	8
Ho	2011	*	*		*	**	*	*		High	7
Song	2012	*	*		*	**	*	*		High	7
Lubbe	2012	*	*		*	**	*	*		High	7
Garcia-Albeniz	2012	*	*		*	**	*	*		High	7
Phipps	2012	*	*		*	**	*	*		High	7
Kirac	2013	*	*	*	*	**	*	*		High	8
Yang	2014	*	*	*		**	*	*		High	7
Kurlapska	2014	*	*	*		**	*	*		High	7
Zhang	2014	*	*	*	*	**		*		High	7
Hong	2015	*	*	*	*	*	*	*		High	7
Baert-Desurmont	2016	*	*		*	**	*	*		High	7
Abd EI-Fattah	2016	*	*		*	**	*	*		High	7
Alonso-Molero	2017	*	*	*		**	*	*		High	7
Shaker	2018	*	*		*	**	*	*		High	7
Reilly	2021	*	*			**	*	*		High	6
Alidoust	2022	*	*		*	**	*	*		High	7
RS4464148											
Broderick	2007	*	*		*	**	*	*		High	7
Thompson	2009	*	*	*	*	**		*		High	7
Curtin	2009	*	*	*	*	**	*	*		High	8
Pittman	2009	*	*		*	**	*	*		High	7
Ho	2011	*	*		*	**	*	*		High	7
Zhang	2014	*	*	*	*	**		*		High	7
Kurlapska	2014	*	*	*		**	*	*		High	7
Damavand	2015	*	*		*	**	*	*		High	7
Serrano-fernadez	2015	*	*	*	*	**	*	*		High	8
Reilly	2021	*	*			**	*	*		High	6
RS12953717											
Broderick	2007	*	*		*	**	*	*		High	7
Middeldorp	2009	*	*		*	*	*	*		High	6
Curtin	2009	*	*	*	*	**	*	*		High	8
Thompson	2009	*	*	*	*	**		*		High	7
Pittman	2009	*	*		*	**	*	*		High	7
Kupfer	2010	*	*		*	**	*	*		High	7
Slattery	2010	*	*		*	**	*	*		High	7
Li	2011	*	*	*	*	**	*	*		High	8
Ho	2011	*	*		*	**	*	*		High	7
Scollen	2011	*	*		*	**	*	*		High	7
Zhang	2014	*	*	*	*	**		*		High	7
Damavand	2015	*	*		*	**	*	*		High	7
Lu	2015	*	*			**	*	*		High	6
Reilly	2021	*	*			**	*	*		High	6

Item 1, Adequate case definition; **Item 2,** Representativeness of the cases; **Item 3,** Selection of controls; **Item 4,** Determination of controls; **Item 5,** Comparability of cases and controls on the design or analysis; **Item 6,** Ascertainment of exposure; **Item 7,** Same method of ascertainment for cases and controls; **Item 8,** Non-response rate; one star (*) indicates 1 point; **High,** studies are regarded as high quality by the NOS.
