# Supplementary material for: Association of several loci of SMAD7 with colorectal cancer: A meta-analysis based on case–control studies
Source: Medicine (Baltimore). 2023 Jan 6;102(1):e32631. doi: 10.1097/MD.0000000000032631 (PMC9829263; doi:10.1097/MD.0000000000032631)
Supplement: Supplementary file 3 [file medi-102-e32631-s003.pdf]

Supplemental Table 3: Results of meta-regression analysis of three polymorphisms.

Covariates	Number of dummy variables	dominant model	recessive model	homozygous model	heterozygous model	additive model
RS4939827						
Ethnicity	4	0. 384	0. 194	0. 125	0. 126	0. 201
Source of controls	4	0. 649	0. 653	0. 568	0. 853	0. 786
Genotyping methods	10	0. 650	0. 083	0. 264	0. 857	0. 196
Sample	2	0. 700	0. 415	0. 563	0. 255	0. 952
Cons		0. 637	0. 104	0. 263	0. 684	0. 269
RS4464148						
Ethnicity	2	0. 420	0. 494	0. 522	0. 373	0. 292
Source of controls	3	0. 496	0. 118	0. 233	0. 725	0. 314
Genotyping methods	8	0. 852	0. 193	0. 507	0. 742	0. 867
Sample	2	0. 256	0. 077	0. 412	0. 051	0. 822
Cons		0. 588	0. 256	0. 537	0. 240	0. 808
RS12953717						
Ethnicity	4	0. 658	0. 028	0. 012	0. 741	0. 455
Source of controls	4	0. 967	0. 637	0. 358	0. 920	0. 331
Genotyping methods	8	0. 993	0. 929	0. 227	0. 662	0. 340
Sample	2	0. 003	0. 455	0. 777	0. 283	0. 326
Cons		0. 07	0. 425	0. 216	0. 516	0. 538

A grid with a red indicates a P value less than 0.05.
