# Supplementary material for: Association of several loci of SMAD7 with colorectal cancer: A meta-analysis based on case–control studies
Source: Medicine (Baltimore). 2023 Jan 6;102(1):e32631. doi: 10.1097/MD.0000000000032631 (PMC9829263; doi:10.1097/MD.0000000000032631)
Supplement: Supplementary file 4 [file medi-102-e32631-s004.pdf]

Supplemental Table 4. Results of quantitative assessment of publication bias.

SNPs	**Dominant model**	**Recessive model**	**Homozygous model**	**Heterozygous model**	**Additive model**
RS4939827	0. 594	0. 597	0. 868	0. 388	0. 774
RS4464148	0. 933	0. 673	0. 797	0. 829	0. 869
RS12953717	0. 433	0. 909	0. 321	0. 395	0. 467
